# Lead-Induced Atypical Parkinsonism in Rats: Behavioral, Electrophysiological, and Neurochemical Evidence for a Role of Noradrenaline Depletion

**DOI:** 10.3389/fnins.2018.00173

**Published:** 2018-03-19

**Authors:** Mariam Sabbar, Claire Delaville, Philippe De Deurwaerdère, Nouria Lakhdar-Ghazal, Abdelhamid Benazzouz

**Affiliations:** ^1^Institut des Maladies Neurodégénératives, UMR 5293, Université de Bordeau, Bordeaux, France; ^2^Centre National de la Recherche Scientifique, Institut des Maladies Neurodégénératives, UMR 5293, Bordeaux, France; ^3^Faculté des Sciences, Equipe Rythmes Biologiques et Environnement, Université Mohammed V, Rabat, Morocco

**Keywords:** lead, Parkinsonism, subthalamic nucleus, globus pallidus, pars reticulata of substantia nigra, noradrenaline, dopamine, electrophysiology

## Abstract

**Background:** Lead neurotoxicity is a major health problem known as a risk factor for neurodegenerative diseases, including the manifestation of parkinsonism-like disorder. While lead is known to preferentially accumulate in basal ganglia, the mechanisms underlying behavioral disorders remain unknown. Here, we investigated the neurophysiological and biochemical correlates of motor deficits induced by sub-chronic injections of lead.

**Methods:** Sprague Dawely rats were exposed to sub-chronic injections of lead (10 mg/kg, i.p.) or to a single i.p. injection of 50 mg/kg *N*-(2-chloroethyl)-*N*-ethyl-2-bromobenzylamine hydrochloride (DSP-4), a drug known to induce selective depletion of noradrenaline. Rats were submitted to a battery of behavioral tests, including the open field for locomotor activity and rotarod for motor coordination. Electrophysiological recordings were carried out in three major basal ganglia nuclei, the subthalamic nucleus (STN), globus pallidus (GP), and substantia nigra *pars reticulata* (SNr). At the end of experiments, post-mortem tissue level of the three monoamines (dopamine, noradrenaline, and serotonin) and their metabolites has been determined using HPLC.

**Results:** Lead intoxication significantly impaired exploratory and locomotor activity as well as motor coordination. It resulted in a significant reduction in the level of noradrenaline in the cortex and dopamine and its metabolites, DOPAC, and HVA, in the striatum. The tissue level of serotonin and its metabolite 5-HIAA was not affected in the two structures. Similarly, DSP-4, which induced a selective depletion of noradrenaline, significantly decreased exploratory, and locomotor activity as well as motor coordination. L-DOPA treatment did not improve motor deficits induced by lead and DSP-4 in the two animal groups. Electrophysiological recordings showed that both lead and DSP-4 did not change the firing rate but resulted in a switch from the regular normal firing to irregular and bursty discharge patterns of STN neurons. Neither lead nor DSP-4 treatments changed the firing rate and the pattern of GP and SNr neurons.

**Conclusions:** Our findings provide evidence that lead represents a risk factor for inducing parkinsonism-like deficits. As the motor deficits induced by lead were not improved by L-DOPA, we suggest that the deficits may be due to the depletion of noradrenaline and the parallel disorganization of STN neuronal activity.

## Introduction

Parkinson's disease (PD) is a neurodegenerative disorder characterized by the manifestation of motor symptoms, which are mainly attributed to the loss of dopaminergic neurons in the substantia nigra pars compacta (SNc) (Ehringer and Hornykiewicz, 1960). Furthermore, PD is not a pure dopaminergic pathology, it is characterized by the additional degeneration of noradrenaline (NA) and serotonin (5-HT) neurons of the locus coeruleus (LC) and the dorsal raphe respectively (Bertrand et al., 1997; Kish, 2003; Fornai et al., 2007).

It is well established that the degeneration of the monoaminergic systems caused a disorganization of the neuronal activity in the subthalamic nucleus (STN), a basal ganglia structure playing a key role in the pathophysiology of PD. Indeed, the regular pattern of the neuronal activity of the STN becomes irregular and bursty in animal models of PD (Bergman et al., 1994; Ni et al., 2001; Belujon et al., 2007; Delaville et al., 2012a) and in PD patients (Hutchison et al., 1998; Benazzouz et al., 2002). It has also been reported that the external and internal globus pallidus (GPe and GPi respectively) neurons exhibited this pathological pattern in PD patients (Hutchison et al., 1994, 1998; Sterio et al., 1994) and in 6-OHDA rat model of PD (Pan and Walters, 1988; Burbaud et al., 1995; Hassani et al., 1996; Ni et al., 2000; Tai et al., 2003). Recently, Delaville et al. (2012a,b) have shown that NA depletion increased the proportion of bursty and irregular neurons in the STN without affecting the neuronal activity of globus pallidus (GP) and the pars reticulata of substantia nigra (SNr).

Epidemiological studies have indicated a potential association between exposure to lead and an increased risk of PD, suggesting a 2–3-fold increase in risk for PD following lead exposure (Duckett et al., 1977; Gorell et al., 1997; Kuhn et al., 1998; Coon et al., 2006). Furthermore, a large case—control study with biomarker data on cumulative exposure to lead, has demonstrated evidence that high cumulative exposure to lead is associated with an increased risk of PD, providing some of the strongest evidence for a role for lead in the development of PD (Weisskopf et al., 2010). To investigate the relationship between this heavy metal and PD, (Jason and Kellogg, 1981) reported that exposure to high levels of lead induced a loss of dopaminergic neurons in the striatum. However, after cessation of exposure, lead brain levels decreased and lead-induced behavioral and neurochemical abnormalities dissipate (Jason and Kellogg, 1981). Recently, in rats, after 3 weeks of lead exposure, no change in the DA tissue content was revealed by HPLC in the striatum (Sabbar et al., 2012). Interestingly, in the same lead-exposed animals, a decrease in the tissue content of NA in the cortex paralleled by a change in the firing pattern of STN neurons were found (Sabbar et al., 2012). However, it is presently unclear if the NA depletion could participate to the atypical Parkinsonism induced by lead intoxication.

In the present study, we investigated the effects of sub-chronic low-dose lead intoxication and NA depletion, induced by a systemic administration of the neurotoxin N-(2-chloroethyl)-Nethyl-2-bromobenzylamine (DSP-4) (Lapiz et al., 2000; Delaville et al., 2012a,b), on locomotor activity and motor coordination, on the tissue content of the three monoamines (dopamine, noradrenaline, and serotonin) and their metabolites, and on the neuronal activity of the three major basal ganglia nuclei, the STN, GP, and SNr.

## Materials and methods

### Animals and housing

Male Sprague Dawley rats (Centre d'Elevage Depré, Saint Doulchard, France) weighing 150–170 g at the time of experiments were used for behavioral and *in vivo* electrophysiological studies. Rats were kept in polycarbonate cages, 3 rats/cage, in a thermostatically controlled room (temperature: 24°C, relative humidity: 45%) on a 12 h-light/12 h-dark schedule with free access to food and water. The body weights of rats were monitored throughout the experiment. All experiments were carried out in strict accordance with the Council Directive 2010/63/EU of the European Parliament and the Council of 22 September 2010 on the protection of animals used for scientific purposes. The experimental protocol was approved by the Ethics local Committee.

### Drugs and solutions

Lead acetate and sodium acetate (Sigma, France) were dissolved in sterile water. L-DOPA (L-3,4-dihydroxyphenylalanine methyl ester hydrochloride), DSP-4 (N-(2-Chloroethyl)-N-ethyl-2- bromobenzylamine hydrochloride), and Benserazide (Sigma, France) were dissolved in saline (0.9%). L-DOPA, the precursor of DA, remains the most effective medication for PD (Tintner and Jankovic, 2002). Benserazide, which is the peripheral decarboxylase inhibitor, was administered to the animals at least 30 min before L-DOPA injection to prevent conversion of L-DOPA to dopamine in the periphery. DSP-4 had neurotoxic actions on noradrenergic neurons and selectively damages noradrenergic terminals originating from the locus coeruleus (LC) (Lapiz et al., 2000). All drugs were dissolved just before use and administrated intraperitoneally. The doses used were 10 mg/kg for lead acetate and sodium acetate, 25 mg/kg for DSP4, 25 mg/kg for benserazide, and 12 mg/kg for L-DOPA.

### Experimental design and groups

**Figure 1:**
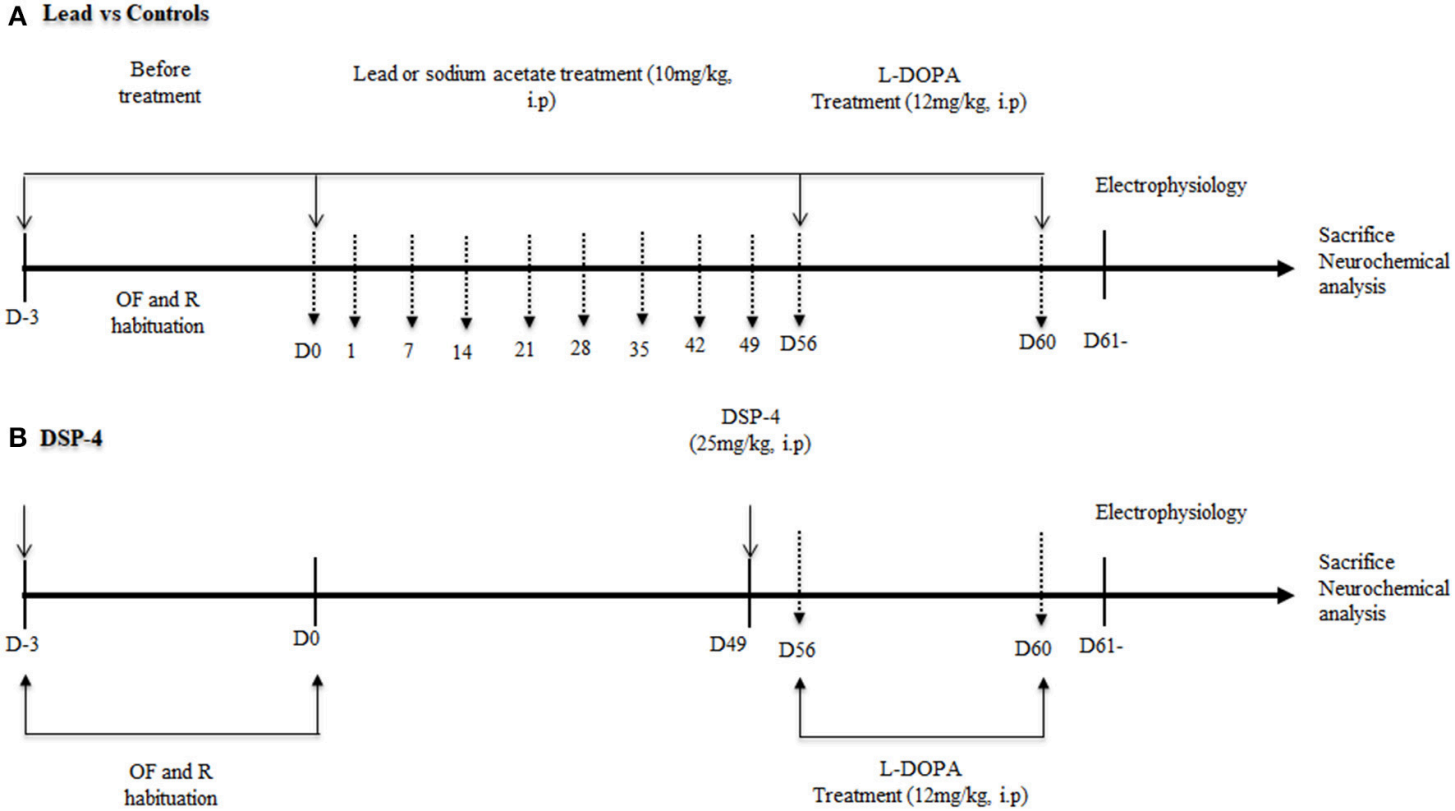
Schematic presentation of the experimental design with a time course of all the behavioral tests (OF: open field, R: rotarod test) and electrophysiological recordings. The vertical hatched arrows correspond to the days when the rats were submitted to the open field and rotarod tests. At the beginning of the study **(A)**, rats were injected with lead acetate or sodium acetate for 56 days. **(B)** One week before the end of lead treatment, another group of rats received an intraperitoneal injection (i.p.) of DSP-4 (25 mg/kg). Lead and DSP-4 rats received a daily i.p. injection of L-DOPA (12 mg/kg) during 4 days. A the end of behavioral tests, rats of each group were processed for electrophysiological recordings in the STN, GP, and SNr. At the end of experiments, the rat brains were processed for post-mortem neurochemical assessment using HPLC.

The experiments were performed as reported in Figure 1. Lead animals (*n* = 18) and controls (*n* = 18) received daily intraperitoneal (i.p.) injection of either lead acetate or sodium acetate, respectively, during 56 days. Behavioral tests were performed every week during treatment. Another group of rats (*n* = 18) received an injection of DSP-4 (Sigma-Aldrich, France) at the dose of 25 mg/kg. DSP-4 solution was administered i.p. once. Behavioral tests were carried out a week after the administration of DSP-4. In a subgroup of lead animals (*n* = 6), L-DOPA was injected i.p. at the end of the lead treatment for 4 days. At day 4, 40 min after L-DOPA injection, locomotor behavior, and motor coordination were evaluated using the open field and the rotarod tests respectively. DSP-4 animals (*n* = 6) received a L-DOPA injection as lead rats and motor behavior was evaluated in the same conditions.

### Behavioral tests

#### Open-field activity test

Exploratory and locomotor activities were evaluated by an “open field” actimeter (Actitrack, Panlab, Barcelona, Spain), which consisted of a transparent cage that was connected to a photoelectric cell. Light beams detected the rat movements and the total locomotor activity of rats of each group was recorded over 20 min. Exploratory and locomotor activities were evaluated as previously described (Sabbar et al., 2012). Behavioral testing in the actimeter was done in an isolated room between 8:00 a.m. and 1:00 p.m. The spontaneous locomotor activity was recorded during 20 min for four consecutive days with habituation during the first 3 days and the test on day 4. The first 5 min session was used to establish the exploratory activity and the last 10 min was used to evaluate locomotor activity. From week 2, behavioral testing was conducted once per week during the 56 days of lead acetate or sodium acetate treatment.

#### Rotarod task

The rotarod test is widely used to evaluate the motor coordination of rodents. The rat was placed on the rod, which consisted of a 7 cm diameter that rotates at different speeds (Bioseb, *in vivo* Research Instruments, Spain). Before the testing sessions, the rats were habituated to stay on the stationary rod for 3 min. Habituation was repeated every day for 3 days before the beginning of lead treatment and the testing sessions. The animal was placed back on the rod immediately after falling, up to 5 times in one session. For testing, the rod was accelerated from 4 to 20 rpm over 180 s and time to fall off the apparatus was recorded for a maximum of 180 s. Two trials were conducted and times were averaged for group comparisons. Testing was conducted once per week during the 56 days of lead acetate or sodium acetate treatment.

### Electrophysiological recordings

Extracellular single-unit recordings were performed as previously described (Ni et al., 2001; Belujon et al., 2007). STN, GP and SNr neurons were recorded in anesthetized rats (urethane 1.2 g/kg, i.p.). After being placed in a stereotaxic instrument, body temperature of rats was maintained at 37°C with a heating pad. All procedures were carried out in accordance with European Communities Council Directive 2010/63/UE to reduce animals suffering. A single glass micropipette electrode was filled with 4% pontamine sky blue in 3 M NaCl, and the electrode tips were broken back under microscopic control until *in vitro* tip impedance measured 8–12 MΩ. Microelectrodes were stereotaxically guided through drilled skull holes to the target coordinates (in mm) for the STN: AP: −3.8, L: −2.5, D: 6.8–8.2; for the GP: AP: −0.9, L: −3, D: 4.5–7.5; and for the SNr: AP: −5.3, L: −2.5, D: 6.5–9 (Paxinos and Watson, 1996). Electrical signals were passed through a preamplifier (Neurolog, Digitimer, UK), and amplified signals were monitored with an oscilloscope and transferred via a Powerlab interface (AD Instruments, Charlotte, NC, USA) to a computer equipped with Chart 5 software (AD Instruments, Charlotte, NC, USA). Only neuronal single-unit activity with a signal-to-noise ratio > 3:1 was recorded. At the end of each recording session, the recording site was marked by electrophoretic injection (Iso DAM 80, WPI, Hertfordshire, UK) of Pontamine sky blue through the micropipette at a negative current of 20 μA for 7 min. The location of the Pontamine sky blue dots was histologically verified as previously reported (Belujon et al., 2007) and only recordings from the brains in which the dot was clearly visible in the STN, GP, and SNr were used for data analysis.

#### Data analysis

The electrical activity of each neuron was analyzed with a spike discriminator using a spike histogram program (AD Instruments, Charlotte, NC, USA). The firing rates and patterns were determined using Neuroexplorer program (AlphaOmega, Nazareth, Israel) and the method developed by Kaneoke and Vitek (1996) as previously described (Labarre et al., 2008).

### Biochemical monoamine assays

After electrophysiological recordings, animals were sacrificed by decapitation, the brains were rapidly removed and dissection of the striatum and the frontal cortex was performed on ice at −20°C. Tissue samples were frozen by immersion in cold isopentane and stored at −80°C until used for neurochemical determinations. Levels of dopamine (DA) and its metabolites, dihydroxyphenylacetic acid (DOPAC), and homovanillic acid (HVA), as well as noradrenaline (NA), serotonin (5-HT), and its metabolite 5 hydroxyindoleacetic acid (5-HIAA) were quantified by a method of high performance liquid chromatography combined with electrochemical detection (HPLC/EC) as previously described (De Deurwaerdere et al., 1995). The samples were weighed, sonicated in 0.1 N perchloric acid, centrifuged 13,000 rpm for 30 min at 4°C. Aliquots of the supernatants were diluted in the mobile phase which contains 60 NaH2PO4, 0.1 mM disodium EDTA, and 2 octane sulfonic acid plus 7% methanol, adjusted to pH 3.9 with orthophosphoric acid. The mobile phase was filtered through a 0.22 μm millipore filter and pumped through the system at a flow rate of 1.2 ml/min. Detection of monoamines and their metabolites was performed with a coulometer detector (CoulochemI, ESA) coupled to a dual-electrode analytic cell (model 5011). The potential of the electrodes was set at +350 and −270 mV.

Concentrations of monoamines were calculated using standard curves. Final results were expressed as means ± SEM in terms of monoamine contents per g of wet tissue (ng/g of tissue). DA and its metabolites (DOPAC and HVA) as well as 5-HT and its metabolite (5-HIAA) were measured in the striatum. NA, 5-HT and its metabolite (5-HIAA) were measured in the frontal cortex of all groups of rats.

### Data processing and statistical analysis

Data are reported as means ± SEM (standard error of the mean). In the present study, sample size was calculated using “resource equation” method as previously reported (Charan and Kantharia, 2013). A value “E” which represents the degree of freedom of analysis of variance (ANOVA) should lie between 10 and 20 and any sample size, which keeps E between these values should be considered adequate. In the present study, we calculated E value using the formula:

$$\begin{array}{l} \text{E }=\text{ Total number of animals}-\text{Total number of groups} \end{array}$$

The E value for all the tests used in the study was largely above 20 indicating that our sample size of animals is adequate.

All statistical procedures were conducted using the GraphPad Prism program (San Diego, USA). Behavioral results were analyzed using a two-way ANOVA comparing several groups. All significant ANOVAs were followed by Sidak's *post-hoc* test. Biochemical and the firing rate data were analyzed using a one-way ANOVA (followed by Tukey's *post-hoc* test). Changes in the proportion of different firing patterns (regular, irregular and bursty) were determined using a Chi^2^ test. The levels of DA, NA, and 5-HT and their respective metabolites were analyzed using one-way ANOVA followed by Tukey's *post-hoc* test when ANOVA was significant. A *P* < 0.05 was considered statistically significant.

## Results

### Body weight changes

**Figure 2:**
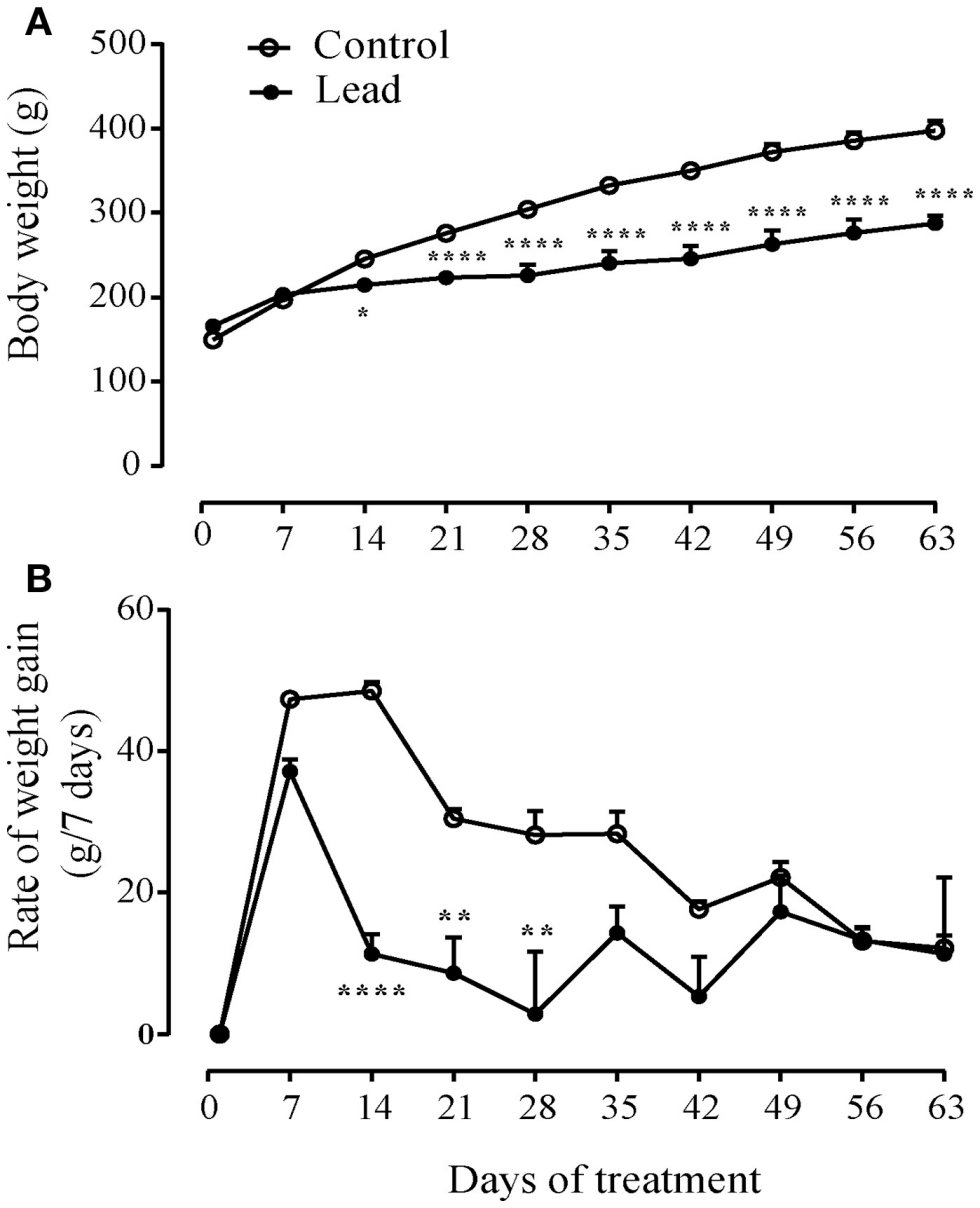
Effect of lead on the evolution of body weight gain. **(A)** lead induced inhibition of body weight gain **(A)** and decreased the rate of body weight gain **(B)** represented as measured at 7 days intervals. Values are the mean ± SEM. Data from lead rats (*n* = 6) and controls (*n* = 6) were compared using the two-way repeated measures ANOVA followed by Sidak's *post-hoc* test. *P < 0.05, **P < 0.01, ****P < 0.0001.

Figure 2A shows that lead acetate injections significantly slowed the progression of body weight gain compared to control rats receiving sodium acetate injections (Two way ANOVA; effect on time [*F*_(9, 45)_ = 250.7, *P* < 0.0001], treatment [*F*_(1, 5)_ = 20.55, *P* = 0.0062] and interaction (treatment × time) [*F*_(9, 45)_ = 22.12, *P* < 0.0001]. At the beginning of the experiment, lead rats had a mean body weight (165.8 ± 7.40 g) that was not significantly different from that of controls (149.5 ± 6.35 g). The two groups of rats grew at similar rates during the first week of treatment. However, within 14 days of treatment, the consequence was the significant reduction in body weight gain in the rats undergoing repeated lead injections (*P* < 0.05), such that at the end of the experiment, the mean body weight of lead rats was significantly lower than that of controls (287.5 ± 9.39 g vs. 397.5 ± 11.53 g, Sidak's *post-hoc* test, *P* < 0.0001, Figure 2A).

Figure 2B shows the mean ± SEM rate of body weight gain from the beginning of the treatment to 1 week after the end of treatment, represented as change in body weight measured at 7 days intervals. All rats were gaining body weight at a rate between 37 and 47 g per week by the time of the first week of the treatment. Two-way repeated measures ANOVA showed significant effects of lead on time [*F*_(9, 45)_ = 15.20, *P* < 0.0001], treatment [*F*_(1, 5)_ = 53.50, *P* = 0.0007] and interaction (treatment × time) [*F*_(9, 45)_ = 4.215, *P* = 0.0005]. Lead treatment caused a significant mean loss of body weight of about 26 g (Figure 2B; two-way ANOVA, followed by Sidak's *post-hoc* test, *P* < 0.01 and *P* < 0.0001). Control rats injected repeatedly with sodium acetate showed a steady growth rate from 21 to about 35 days during the treatment. Peak rate of growth was 48.5 ± 1.33 g/7 days. Thereafter, the rate of growth decreased in all rats, presumably as they approached adult rat body weight.

### Effect of lead and DSP-4 treatments on exploratory and locomotor activities

**Figure 3:**
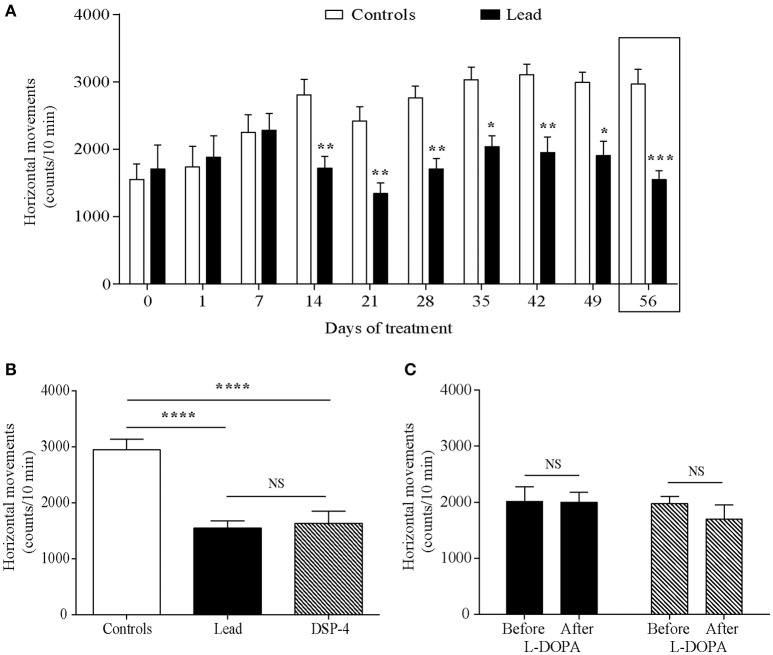
Effects of lead, DSP-4 and L-DOPA treatment on exploratory locomotor activity. Exploratory activity histograms represent the number of horizontal movements recorded during the first session of 5 min in the “open field” before and during lead treatment **(A)**. **(B)** The number of horizontal movements recorded during 5 min on day 56 of lead treatment was compared with the exploratory activity of DSP-4 treated animals. **(C)** Histograms showing the effect of L-DOPA treatment in lead and DSP-4 rats. Values are the mean ± SEM. Data from controls (*n* = 12) and lead rats (*n* = 12) on **(A)** were compared using the two-way ANOVA followed by Sidak's *post-hoc* test. Data from controls, lead and DSP-4 rats (*n* = 12) on **(B)** were compared using the one-way ANOVA followed by Tukey's *post-hoc* test. Data from lead and DSP-4 rats before and after L-DOPA treatment on **(C)** were compared using the Mann Whitney test. *P < 0.05, **P < 0.01, ***P < 0.001, ****P < 0.0001, NS no significant.

**Figure 4:**
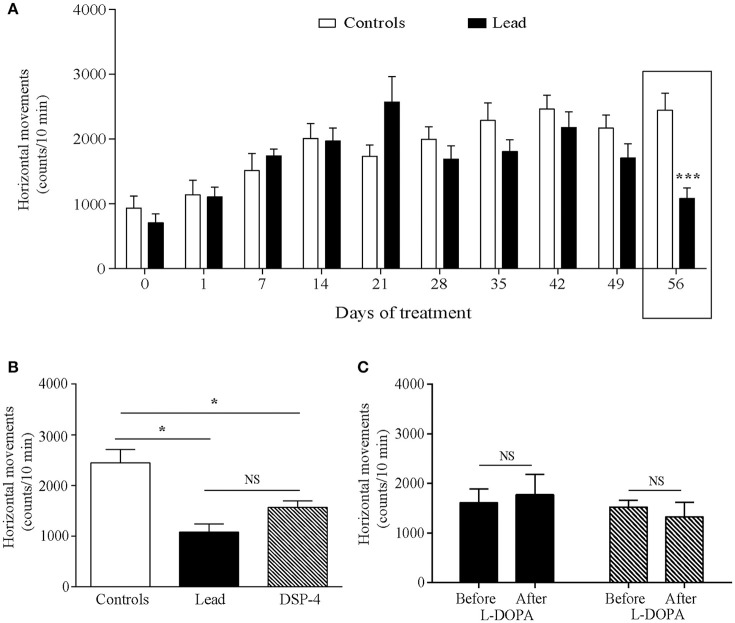
Effects of lead, DSP-4 and L-DOPA treatment on locomotor activity. Locomotor activity histograms represent the number of horizontal movements recorded during the last session of 10 min in the “open field” before and during lead treatment **(A)**. **(B)** The number of horizontal movements recorded during 10 min on day 56 in lead rats was compared with the locomotor activity of controls and DSP-4 treated animals. **(C)** Histograms showing the effect of L-DOPA treatment in lead and DSP-4 rats. Values are the mean ± SEM. Data from controls and lead rats on **(A)** were compared using the two-way ANOVA followed by Sidak's *post-hoc* test. Data from controls (*n* = 12), lead (*n* = 12) and DSP-4 (*n* = 10) rats on **(B)** were compared using the one-way ANOVA followed by Tukey's *post-hoc* test. Data from lead and DSP-4 rats before and after L-DOPA treatment on **(C)** were compared using the Mann-whitney test. *P < 0.05, ***P < 0.001, NS, no significant.

Horizontal movements measured during the first 5 min in the actimeter were used to assess the rats' exploratory activity and those measured during the last 10 min were considered as the rats' locomotor activity. Two-way ANOVA analysis showed that lead significantly affected the exploratory activity in the open field [*F*_(1, 214)_ = 56.70, *P* < 0.0001, Figure 3A] with an interaction between lead treatment and time [*F*_(9, 214)_ = 3.725, *P* = 0.0002]. Sidak's *post-hoc* test showed that lead rats significantly reduced the number of their horizontal movements during the first 5 min-testing sessions, which started 14 days after the beginning of the treatment and remained lower at day 56 of lead treatment, when compared to control rats. In contrast to exploratory activity, two-way ANOVA analysis of the locomotor activity showed a delayed, but significant, effect of lead treatment on locomotor activity recorded during the last 10 min of each session [*F*_(1, 214)_ = 4.639, *P* = 0.0324, Figure 4A] as well as of lead treatment × sessions interaction [*F*_(9, 214)_ = 3.167, *P* = 0.0013]. Compared to control rats, the significant decrease in locomotor activity has been observed only at day 56 of lead treatment (Sidak's *post-hoc* test, *P* < 0.01, Figure 4A).

The effects of DSP-4 on the exploratory and locomotor activities were evaluated at day 56 and compared to the effect of lead treatment at the same time point. DSP-4 rats showed a decrease in their exploratory and locomotor activities as expressed by the significant reduction in the number of horizontal movements measured during the first 5 min session [One way ANOVA, *F*_(2, 29)_ = 19.04; *P* < 0.0001 followed by Tukey's *post-hoc* test, *P* < 0.0001; Figure 3B] and the last 10 min session [One way ANOVA, *F*_(2, 29)_ = 11.85; *P* = 0.0002 followed by Tukey's *post-hoc* test, *P* < 0.05; Figure 4B] in the open field test compared to controls. When compared to lead rats, no significant difference was observed.

The 4 days of L-DOPA treatment did not improve either the exploratory (*P* > 0.05, Figure 3C) or the locomotor (*P* > 0.05, Figure 4C) activities measured in the actimeter in lead rats as well as in DSP-4 rats.

### Effect of lead and DSP-4 treatments on motor coordination

**Figure 5:**
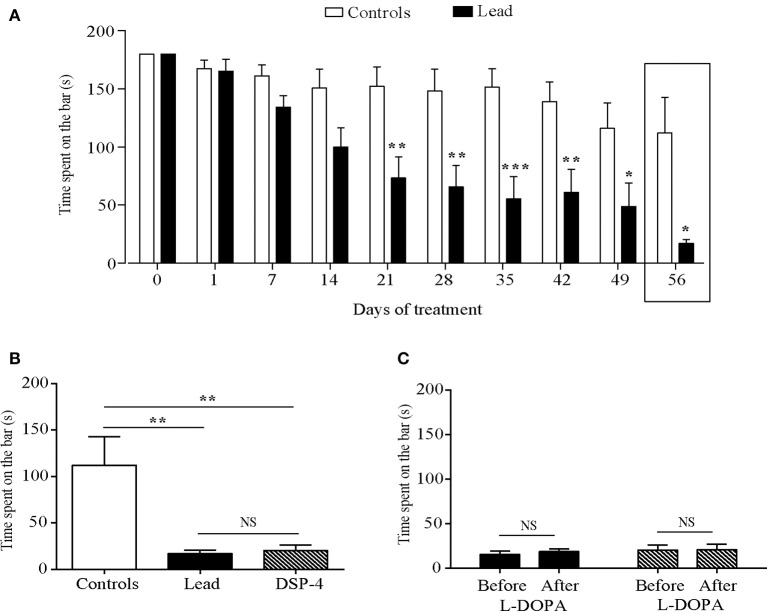
Effects of lead, DSP-4 and L-DOPA treatment on motor coordination assessed by the rotarod test. **(A)** Motor coordination histograms represent the time spent on the rotarod bar in control and lead treated rats. **(B)** Histograms showing the comparison of the time spent on the rotarod bar in control, lead and DSP-4 rats on day 56. **(C)** Histograms showing the effect of L-DOPA in lead and DSP-4 rats. Values are the mean ± SEM. Data from controls and lead rats on **(A)** were compared using the two-way ANOVA followed by Sidak's *post-hoc* test. Data from controls (*n* = 6), lead (*n* = 6), and DSP-4 rats (*n* = 6) on **(B)** were compared using the one-way ANOVA followed by Tukey's *post-hoc* test. Data from lead (*n* = 6) and DSP-4 (*n* = 6) rats on **(C)** were compared using the Mann whitney test. *P < 0.05, **P < 0.01, ***P < 0.001, NS, no significant.

Lead treatment affected motor coordination as shown by the decrease in the time spent on the rotated bar in the rotarod test [two-way ANOVA: effect on time *F*_(9, 181)_ = 9.616, *P* < 0.0001, treatment, *F*_(1, 181)_ = 63.51, *P* < 0.0001, and treatment × time interaction, *F*_(9, 181)_ = 2.551, *P* = 0.0088). Lead rats spent less time on the bar compared to the controls since day 22 (*P* < 0.05; *P* < 0.01, *P* < 0.001, Sidak's *post-hoc* test; Figure 5A).

One way ANOVA analysis showed that lead as well as DSP-4 treatments significantly decreased the time spent on the rotated bar compared to controls at day 56 [*F*_(2, 15)_ = 8.796, *P* < 0.0030, Figure 5B]. DSP-4 treated animals showed a significant decrease of the time spent on the rotated bar compared to controls (*P* < 0.01, Figure 5B). The effect of DSP-4 was similar to that of lead-treated rats, as no significant difference was observed between the two groups (*P* > 0.05, Figure 5B) and to lead treated animals (*P* < 0.01 and *P* < 0.01 respectively, Figure 5B).

L-DOPA treatment did not improve the motor coordination in lead rats as well as in DSP-4 rats as it did not improve the time spent on the rotated bar in the two groups (*P* > 0.05, Figure 5C).

### Effects of lead and DSP-4 on the neuronal activity of STN, GP, and SNr

#### Effects on STN neurons

**Figure 6:**
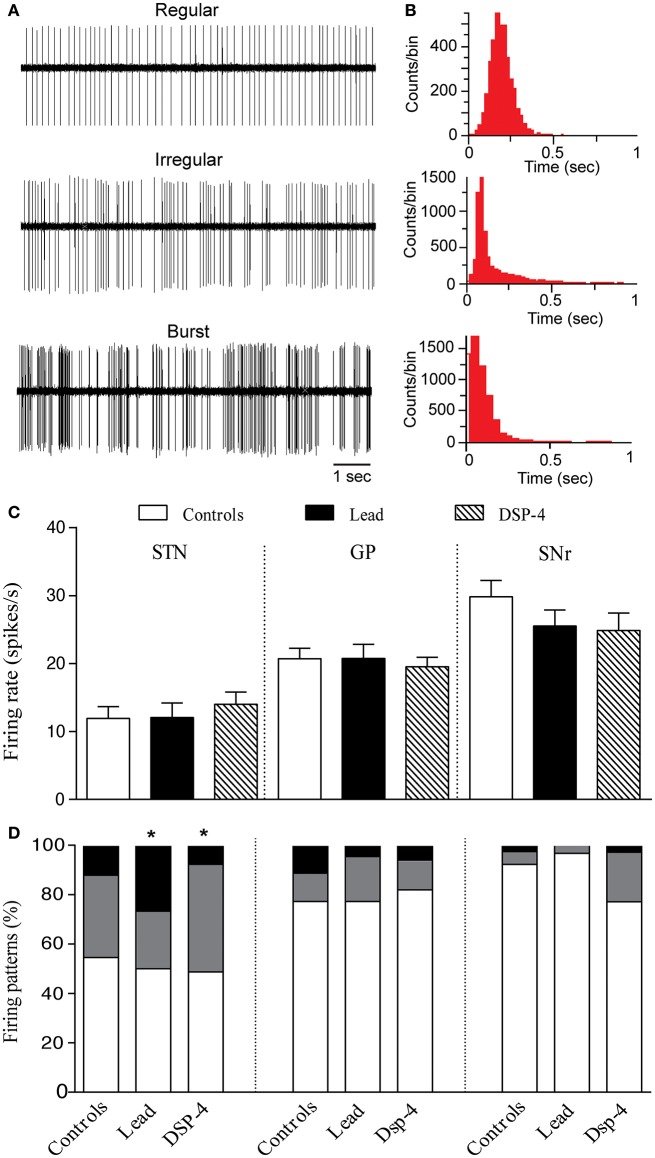
Effects of lead and DSP-4 treatments on the electrical activity of STN, GP, and SNr neurons. **(A)** Representative examples of spike trains and the corresponding interspike interval histograms **(B)** showing regular, irregular and bursty neurons recorded in the STN. **(C)** Histograms represent the mean ± SEM of the firing rate of all neurons recorded in each experimental group (control, lead, and DSP-4 rats). Firing rate data from control, lead and DSP-4 rats were compared using the one-way ANOVA. **(D)** Firing pattern histograms showing the proportion (%) of STN, GP and SNr cells discharging regularly (white portion), irregularly (gray portion) or with bursts (black portion). Changes in the proportion of different firing patterns were analyzed using a Chi^2^ test. *P < 0.05 comparison with controls.

A total of 102 neurons were recorded in the STN of control, lead and DSP-4 rats. The firing rate of STN neurons was not significantly different in the three different groups of animals [One way ANOVA, *F*_(2, 99)_ = 0.4103, *P* = 0.66, Figures 6A,C] as previously reported (Delaville et al., 2012a; Sabbar et al., 2012). In control rats, the mean firing rate of STN neurons was 11.92 ± 1.74 spikes/s (*n* = 33), in lead treated rats 12.04 ± 2.15 spikes/s (*n* = 30) and in DSP-4 rats 14.01 ± 1.81 spikes/s (*n* = 39).

Concerning the firing pattern, sub-chronic lead treatment significantly increased the proportion of STN neurons with bursty pattern compared to controls (Chi^2^ test, *X*^2^ = 13.083, *df* = 2, *P* = 0.010, Figures 6B,D). In DSP-4 rats, a significant increase in the proportion of irregular neurons was found compared to controls (Chi^2^ test, *X*^2^ = 13.083, *df* = 2, *P* = 0.010; Figure 6B).

#### Effects on GP neurons

A total of 138 neurons were recorded in the GP of control, lead and DSP-4 rats. Neither the lead treatment nor the DSP-4 treatment affected the firing rate of GP neurons [One way ANOVA, *F*_(2, 135)_ = 0.1738, *P* = 0.84, Figure 6A]. The firing rate of GP neurons was 20.70 ± 1.57 spikes/s (*n* = 33) in control rats, 20.75 ± 2.08 spikes/s (*n* = 30) in lead treated rats and 19.53 ± 1.38 spikes/s (*n* = 39) in DSP-4 rats. Furthermore, the firing pattern of GP neurons was also unaffected by either lead or DSP-4 treatments (Chi^2^ test, *X*^2^ = 0.977, *df* = 2, *P* = 0.61 for lead rats, and Chi^2^ test, *X*^2^ = 5.181, *df* = 2, *P* = 0.075 for DSP-4 rats when compared to controls, Figure 6B).

#### Effects on SNr neurons

We examined also the extracellular single-unit activity of SNr neurons and we recorded a total of 108 neurons in control, lead and DSP-4 rats. The firing rate of SNr neurons was not significantly different in the three different groups [One way ANOVA, *F*_(2, 105)_ = 1.259, *P* = 0.29, Figure 6A]. In control rats, the mean firing rate of SNr neurons was 29.82 ± 2.44 spikes/s (*n* = 33), in lead treated rats 25.51 ± 2.38 spikes/s (*n* = 30) and in DSP-4 rats 24.88 ± 2.56 spikes/s (*n* = 39). Furthermore, either lead treatment nor DSP-4-induced NA depletion affected the firing pattern of SNr neurons (Chi^2^ test, *X*^2^ = 5.934, *df* = 2, *P* = 0.051 for lead rats, and Chi^2^ test, *X*^2^ = 2.594, df = 2, *P* = 0.27 for DSP-4 rats when compared to controls, Figure 6B).

### Effect of lead and DSP-4 on monoamine tissue levels in the striatum and the frontal cortex

Lead as well as DSP-4 treatments significantly affected the tissue level of NA in the frontal cortex [One-way ANOVA, *F*_(2, 18)_ = 6.741, *P* = 0.0065, Table 1]. Indeed, lead treatment significantly decreased the tissue level of NA in the cortex (−25.5%, *P* < 0.05, Table 1) as DSP-4 did (−39.5%, *P* < 0.01, Table 1) in comparison to controls. However, lead but not DSP-4, decreased the tissue level of DA (−64.6%, *P* = 0.0006) and its metabolites DOPAC (−66.2%, *P* < 0.0001) and HVA (−65.4%, *P* = 0.0105) in the striatum. Furthermore, neither lead nor DSP-4 affected the tissue contents of 5-HT and 5-HIAA in the striatum [5-HT: One way ANOVA, *F*_(2, 18)_ = 0.089, *P* = 0.91; 5-HIAA: One way ANOVA, *F*_(2, 18)_ = 0.041, *P* = 0.96] and in the cortex (5-HT: One way ANOVA, *F*_(2, 18)_ = 1.458, *P* = 0.26; 5-HIAA: One way ANOVA, *F*_(2, 18)_ = 3.080, *P* = 0.07).

**Table 1:** Neurochemical analysis showing the tissue level of DA, NA, and 5-HT as well as their metabolites (DOPAC and HVA for DA, and 5-HIAA for 5-HT) in control, lead and DSP-4 rats.

**Animal groups**	**Striatum (ng/g of tissue)**					**Frontal cortex (ng/g of tissue)**		
	**DA**	**DOPAC**	**HVA**	**5-HT**	**5-HIAA**	**NA**	**5-HT**	**5-HIAA**
Controls	5939 ± 1001	977 ± 144	1134 ± 226	158 ± 52	424 ± 102	226 ± 13	331 ± 21	217 ± 26
Lead	2128 ± 394^**^	330 ± 30^**^	392 ± 60^**^	180 ± 54	392 ± 86	142 ± 21^*^	250 ± 40	173 ± 19
DSP-4	5480 ± 435^++^	1013 ± 84^+++^	752 ± 113	186 ± 37	424 ± 75	137 ± 16^**^	263 ± 28	151 ± 15

Lead treatment induced selective depletion of NA and DA and its metabolites DOPAC and HVA without affecting the tissue content of 5-HT and 5-HIAA. Values are tissue contents in ng/g of wet tissue presented as the mean ± SEM. Data from controls (n = 6), lead (n = 6) and DSP-4 (n = 10) rats were compared using the one-way ANOVA followed by Tukey's post-hoc test.

* P < 0.05,

** P < 0.01 comparison of controls vs. lead rats and controls vs. DSP-4 rats.

++ P < 0.01,

+++ *P < 0.001 comparison of lead vs. DSP-4 rats*.

## Discussion

It has been reported that lead exposure can cause neurotoxicity and neurologic disorders that resembles PD. The present study is an extension of our previous work (Sabbar et al., 2012), and to our knowledge, our data provide evidence that lead intoxication induced atypical Parkinsonism that can be differentiated from PD. Here, we used a subchronic treatment of lead at the dose of 10 mg/kg, which induced a decrease in DA as well as NA tissue contents in contrast to our previous work (Sabbar et al., 2012), in which we showed that the dose of 20 mg/kg induced a decrease in NA tissue content only, without affecting the level of DA. These depletions resulted in a decrease of exploratory and locomotor activities as well as motor coordination, which were paralleled by the disorganization of the firing pattern of STN neurons without affecting the electrophysiological parameters of GP and SNr neurons. Furthermore, similar behavioral and electrophysiological results have been found after the treatment with DSP4-induced NA depletion. To confirm the atypical character of Parkinsonism induced by lead and DSP-4, we tested the behavioral effect of L-DOPA, and our data are the first to show the ineffectiveness of this dopaminergic antiparkinsonian agent on motor disabilities induced by the two neurotoxicants.

Lead is a well-established neurotoxicant heavy metal, and a growing body of data suggested that lead neurotoxicity is associated with elevated risk of PD (Duckett et al., 1977; Gorell et al., 1997; Kuhn et al., 1998; Coon et al., 2006). Thus, in the present study, were investigated the relationship between this heavy metal and Parkinsonism.

Two weeks after a recurrent i.p. administration of 10 mg/kg of lead acetate, the treated rats showed an inhibitory effect on body weight gain and a reduced ability of rate of growth as previously reported in lead-treated pregnant rats and pups (Aprioku and Siminialayi, 2013). This effect could be attributed, at least in part, to a deficiency in energy metabolism and to the alterations of normal cell metabolism (Patel et al., 1974; Wapnir et al., 1977). More specifically, it could be attributed to lead-induced zinc deficiency (Bushnell and Levin, 1983; Taha et al., 2013). Moreover, the reduction of body weight gain can be due to a reduction in food intake as it has been shown in kids that clinical symptoms of lead exposure generally begin with loss of appetence (Lowry, 2010). Since we did not measure food intake in lead group, and there was no apparent change in food consumption in our lead rats, we can only speculate that there is metabolic dysfunction. Indeed, one of the effects of lead exposure is on glutathione metabolism (Hunaiti et al., 1995). Glutathione is an important antioxidant for quenching free radicals in the liver and glutathione metabolism considered as a highly effective compensatory mechanism to overcome metal toxicity (Hsu, 1981). However, this mechanism seems no longer effective following a long-term lead toxicity as a decrease in glutathione reductase, glutathione peroxidase, and glutathione-S-transferase levels were observed in occupationally-exposed workers that are correlated with depressed glutathione (Adonaylo and Oteiza, 1999) leading to reduce body weight gain.

Sub-chronic lead intoxication resulted in a decrease of exploratory and locomotor activities as measured in the open field test. These locomotor impairments associated with the low exploratory behavior are consistent with previous studies (Reiter et al., 1975; Moreira et al., 2001; NourEddine et al., 2005; Reckziegel et al., 2011; Sansar et al., 2011). It might be argued that the low exploratory behavior is due to a partial lack of motivation of lead-treated rats to explore the open field arena. However, it is unlikely to consider this hypothesis as in a previous study we have shown that lead did not induce “depressive-like” disorder (Sabbar et al., 2012).

Furthermore, lead rats displayed an impairment in the rotarod performance, which accounts for deficits in motor coordination and balance (Hamm et al., 1994; Rogers et al., 1997; Rozas et al., 1997). These results are in line with our previous study (Sabbar et al., 2012), in which we showed that lead intoxication at the dose of 25 mg/kg during 3 weeks altered NAergic transmission that caused motor impairments. Indeed, i.p. injection of DSP-4 (25 mg/kg), a neurotoxin which affects primarily NA terminals arising from the LC (Jonsson et al., 1982; Fritschy and Grzanna, 1989; Fritschy et al., 1990), induced low exploratory behavior, hypolocomotor activity, and impaired motor coordination. In line with earlier reports (Berridge and Dunn, 1990; Harro et al., 1995; Delaville et al., 2012a; Sabbar et al., 2012), our results add strong evidence that the observed exploratory and motor disturbances following lead treatment are due to the depletion of NA.

Although the NAergic system is rarely associated with motor functions, a growing number of data strengthened the involvement of abnormal NAergic neurotransmission in mediating motor disabilities (see review Rommelfanger et al., 2007; Delaville et al., 2011, 2012a; Pifl et al., 2013). Indeed, Rommelfanger et al. (2007) showed that knockout mice (Dbh−/−) that lack NA display robust motor deficits including motor coordination impairment. Another study by Delaville et al. (2012a) reported similar motor impairment after DSP-4 treatment in the rat. In addition, in PD, besides the DA neurodegeneration (Ehringer and Hornykiewicz, 1960), it has been shown that PD is also characterized by the degeneration of NA neurons in the LC (Forno, 1996; Bertrand et al., 1997), which is greater than the degeneration of DA neurons in the SNc (Zarow et al., 2003).

Our results showed that 4 days of L-DOPA treatment failed to improve motor disabilities in lead as well as in DSP-4 rats. Because L-DOPA has been shown to be highly effective in the alleviation of motor disabilities in animal models of PD and PD (Navailles et al., 2010, 2014; Nevalainen et al., 2014; De Deurwaerdère et al., 2017), our results suggest that the motor deficits induced by lead and DSP-4 correspond to atypical Parkinsonism. Indeed, at the dose used in our study, L-DOPA has been shown to enhance extracellular levels of DA in lesioned and non-lesioned rats (Navailles et al., 2010; Nevalainen et al., 2014). Its effect is likely preserved in our model due to the lack of alteration of serotonergic neurons which are responsible for L-DOPA-induced DA release (Navailles et al., 2010), and would be even magnified with the loss of NA terminals (Navailles et al., 2014). Thus, the severe drop of neurochemical dopaminergic markers in the striatum (>60% decrease for DA tissue content and its metabolites) is unlikely responsible for the parkinsonism induced by lead. L-DOPA is also a 2-step metabolic precursor of NA but its effects on NA release are modest and still a matter of debate (De Deurwaerdère et al., 2017). It could not be sufficient to counteract the reduction of NA tissue contents induced by DSP-4 and lead, presumably due to a destruction of NAergic terminals. Additional experiments with pharmacological agents rescuing NAergic transmission more selectively than L-DOPA are warranted. Our data confirm that lead intoxication alters DAergic and NAergic neurotransmission (Jason and Kellogg, 1981; Lorton and Anderson, 1986; Albin et al., 1989; Selvín-Testa et al., 1994; Thach, 1998; Tavakoli-Nezhad et al., 2001) and stress a possible role of NA deficit in the atypical Parkinsonism we report.

A large number of investigations reported that lead neurotoxicity affected several brain areas that are involved in motor control, such as the cerebral cortex and basal ganglia (Tavakoli-Nezhad et al., 2001; Sabbar et al., 2012). This may underlie, at least in part, the motor deficits observed in the present study. However, it should be considered that brain regions other than the basal ganglia can be involved in the long-term motor disabilities produced by lead, including the cerebellum, a region playing a critical role in motor coordination, and balance (Rozas et al., 1997; Thach, 1998), reported to be affected after lead intoxication (Lorton and Anderson, 1986; Selvín-Testa et al., 1994).

The basal ganglia are a group of highly interconnected subcortical nuclei that are involved in motor control (Alexander et al., 1986; Albin et al., 1989; Temel et al., 2006). According to the basal ganglia functional model, the striatum represents the main input structure of the system and SNr and the entopeduncular nucleus (GPi, internal GP in primate) represent the output structures projecting mainly to the thalamus. Input and output structures are linked by a monosynaptic direct pathway and polysynaptic indirect pathway that involves GP (GPe, external GP in primate) and the STN (Albin et al., 1989; Alexander and Crutcher, 1990; Alexander et al., 1990; Burkhardt et al., 2007). Studies that have investigated the pathophysiology of PD have provided significant insight into the complex role of DA and NA depletions on the neuronal activity of basal ganglia structures (Delaville et al., 2012a,b). Indeed, STN neurons, which normally exhibit a tonic discharge pattern, become irregular, and bursty in animal models of PD (Bergman et al., 1994; Ni et al., 2001; Delaville et al., 2012a). The pathological pattern has also been reported in the STN, GPe/GPi of PD patients (Hutchison et al., 1994, 1998; Sterio et al., 1994; Benazzouz et al., 2002).

In the current study, our electrophysiological results show that chronic lead treatment did not affect the firing rate of STN neurons, in line with several previous studies where investigators were unable to detect changes in the firing rate of STN neurons in the rat model of PD (Ni et al., 2001; Tai et al., 2003; Belujon et al., 2007; Delaville et al., 2012a) and in lead-induced neurotoxicity in rats (Sabbar et al., 2012). Interestingly, DSP-4 treatment, when combined with 6-OHDA injection, did not affect the firing rate of STN neurons (Delaville et al., 2012a). Nevertheless, it should be noted that unlike the firing rate, the firing pattern is considered as the most relevant and consistent parameter in the pathophysiology of PD (Bergman et al., 1994; Ni et al., 2001; Tai et al., 2003; Meissner et al., 2005; Belujon et al., 2007; Chetrit et al., 2013). Accordingly, our results show that lead treatment induced a profound disorganization in the firing pattern of STN neurons. Indeed, NA depletion induced by DSP-4 injection provoked a switch from regular to irregular pattern whereas lead treatment induced a switch from regular to bursty and irregular pattern compared to controls. The latter results are in agreement with those observed by Delaville et al. (2012a) in which DSP-4 treatment in rats significantly increased the proportion of irregular and bursty neurons in the STN. Interestingly, and in contrast with earlier studies carried out in 6-OHDA rat model of PD (Pan and Walters, 1988; Burbaud et al., 1995; Hassani et al., 1996; Ni et al., 2000; Tai et al., 2003), we did not observe any change in the electrophysiological parameters of GP and SNr neurons following lead treatment or DSP-4 administration. These results suggest that the behavioral changes observed after lead intoxication are due to the depletion of DA, which is responsible of the changes in the STN neuronal activity without affecting GP and SNr neurons. Our results fit with those of Delaville et al. (2012b), who also showed that DSP-4 injections did not affect either the firing rate or the firing pattern of GP and SNr neurons. Nevertheless, the same authors have also reported that 6-OHDA/DSP-4 injections affected the firing rate and the firing pattern of SNr neurons (Delaville et al., 2012b). These results are different from those observed in rats intoxicated with lead in whom both DA and NA depletions occurred. These discrepancies can be explained by the fact that the observed DA/NA depletions in lead rats did not reach the critical level to induce electrophysiological changes in the SNr as reported in the 6-OHDA rat model of PD.

In our study we focused on the consequences of lead intoxication on brain functions. However, lead toxicity is multifactorial and it is unlikely that its effects are limited to brain functions. In addition to the central and peripheral nervous system, lead toxicity has shown many harmful effects. It is known to induce a broad range of physiological, biochemical and behavioral dysfunctions in humans and lab. animals, including hematopoietic system, cardiovascular system, kidneys, liver, and reproductive system (Matović et al., 2015; Pal et al., 2015).

In summary, our findings provide strong evidence that lead-induced atypical Parkinsonism expressed by the impairment of exploratory and locomotor activities as well as motor coordination. These disorders are associated principally with the depletion of NA, with a low implication of DA depletion. These two monoamines are known to be involved in motor functions. As described in several reports about the pathophysiology of PD (Forno, 1996; Bertrand et al., 1997), it is possible that lead neurotoxicity affected first the NAergic system (Sabbar et al., 2012) before the DAergic system, and that the depletion of NA is essential in the manifestation of atypical Parkinsonian-like motor disabilities, which were paralleled by the irregular/busrty firing pattern of STN neurons. Our study highlights and reinforces the possible contribution of lead, as an environmental factor, in the development of atypical Parkinsonism. Further experiments are needed to determine the direct link between lead intoxication, the depletion of NA and the behavioral impairments and electrophysiological changes.

## Author contributions

MS: carried out the experiments, collected, and analyzed the data, wrote and edited the manuscript; AB: designed the experimental protocol, supervised the work, and participated in writing the paper; CD: assisted with data collection; PD: supervised the neurobiochemical analysis of monoamines; NL-G and AB: edited and approved the final draft of the manuscript. All authors read and approved the final manuscript.

### Conflict of interest statement

The authors declare that the research was conducted in the absence of any commercial or financial relationships that could be construed as a potential conflict of interest.
